# Non-junctional Cx32 mediates anti-apoptotic and pro-tumor effects via epidermal growth factor receptor in human cervical cancer cells

**DOI:** 10.1038/cddis.2017.183

**Published:** 2017-05-11

**Authors:** Yifan Zhao, Yongchang Lai, Hui Ge, Yunquan Guo, Xue Feng, Jia Song, Qin Wang, Lixia Fan, Yuexia Peng, Minghui Cao, Andrew L Harris, Xiyan Wang, Liang Tao

**Affiliations:** 1 Department of Pharmacology, Zhongshan School of Medicine, Sun Yat-Sen University, Guangzhou 510080, China; 2 Department of Anesthesiology, Sun Yat-Sen Memorial Hospital, Sun Yat-Sen University, Guangzhou 510120, China; 3 Tumor Research Institute, Xinjiang Medical University Affiliated Tumor Hospital, Urumqi, Xinjiang 830000, China; 4 Department of Pathology, Xinjiang Medical University Affiliated Tumor Hospital, Urumqi, Xinjiang 830000, China; 5 Department of Pharmacology, Physiology and Neuroscience, New Jersey Medical School - Rutgers University, Newark, NJ 07103, USA

## Abstract

The role of connexin proteins (Cx), which form gap junctions (GJ), in progression and chemotherapeutic sensitivity of cervical cancer (CaCx), is unclear. Using cervix specimens (313 CaCx, 78 controls) and CaCx cell lines, we explored relationships among Cx expression, prognostic variables and mechanisms that may link them. In CaCx specimens, Cx32 was upregulated and cytoplasmically localized, and three other Cx downregulated, relative to controls. Cx32 expression correlated with advanced FIGO staging, differentiation and increased tumor size. In CaCx cell lines, Cx32 expression suppressed streptonigrin/cisplatin-induced apoptosis in the absence of functional GJ. In CaCx specimens and cell lines, expression of Cx32 upregulated epidermal growth factor receptor (EGFR) expression. Inhibition of EGFR signaling abrogated the anti-apoptotic effect of Cx32 expression. In conclusion, upregulated Cx32 in CaCx cells produces anti-apoptotic, pro-tumorigenic effects *in vivo* and *vitro*. Abnormal Cx32 expression/localization in CaCx appears to be both a mechanism and biomarker of chemotherapeutic resistance.

Connexin proteins (Cx) compose vertebrate gap junctions (GJ), which modulate essential cellular processes including electrical coupling, proliferation, differentiation and apoptosis.^1, 2^ Consistent with the idea of ‘contact growth inhibition’ originally proposed in the 1960s,^3, 4^ GJ and Cx have been widely accepted as tumor suppressive; loss of GJ is characteristic of malignancy.^5, 6, 7^ In malignant tumor cells, transfection with Cx enhances radiotherapy/chemotherapy-induced apoptosis in a GJ-dependent manner (toxic ‘bystander effect’).^1, 8, 9^ Our recent work demonstrated that GJ facilitated cisplatin-induced apoptosis in cancerous cells, but suppressed apoptosis in normal cells.^10, 11^ Besides, other studies have shown that expression of Cx can induce cancer cell growth inhibition and apoptosis independent of GJ function.^12, 13, 14^ It was also reported that cytoplasmic Cx exerts pro-tumor effects during metastasis in many cancers, including colorectal, gastric, breast, prostate and liver,^15, 16, 17, 18, 19^ and conducts chemoresistance in glioblastoma.^20, 21^ In contrast, it was recently reported that Cx suppressed metastasis of liver cancer cells.^22^ Therefore, the role of Cx expression, independent of GJs, in cancer pathogenesis is still controversial.

Worldwide, morbidity and mortality of cervical cancer (CaCx) rank fourth in cancers of women.^23, 24^ It is well accepted that human papillomavirus (HPV) infection is highly correlated with cervical cancer.^25, 26^ Loss of GJIC and reduction of expression of Cx26, Cx30 and Cx43 were described in HPV-infected dysplastic cervical epithelial cells.^27, 28^ However, these reports are based primarily on *in vitro* or animal experiments; data on expression and distribution of Cx in human CaCx specimens are rare. The relationships among Cx expression, GJ function and carcinogenesis in CaCx are still largely unknown.

The present study was designed to reveal the role and mechanism of Cx expression in CaCx. A clinical-pathological investigation of four Cx isoforms (Cx26, Cx30, Cx32, and Cx43) was performed to detect their expression and distribution in normal cervix specimens and multiple grades of CaCx. The main goal was to explore the contribution of Cx expression to pathogenesis of CaCx to expand diagnostic and therapeutic options. We also provide empirical evidence for a mechanism by which Cx expression may promote chemoresistance in CaCx.

## Results

### Unlike other connexins, Cx32 is aberrantly upregulated and mislocalized in human CaCx tissue

**Figure 1:**
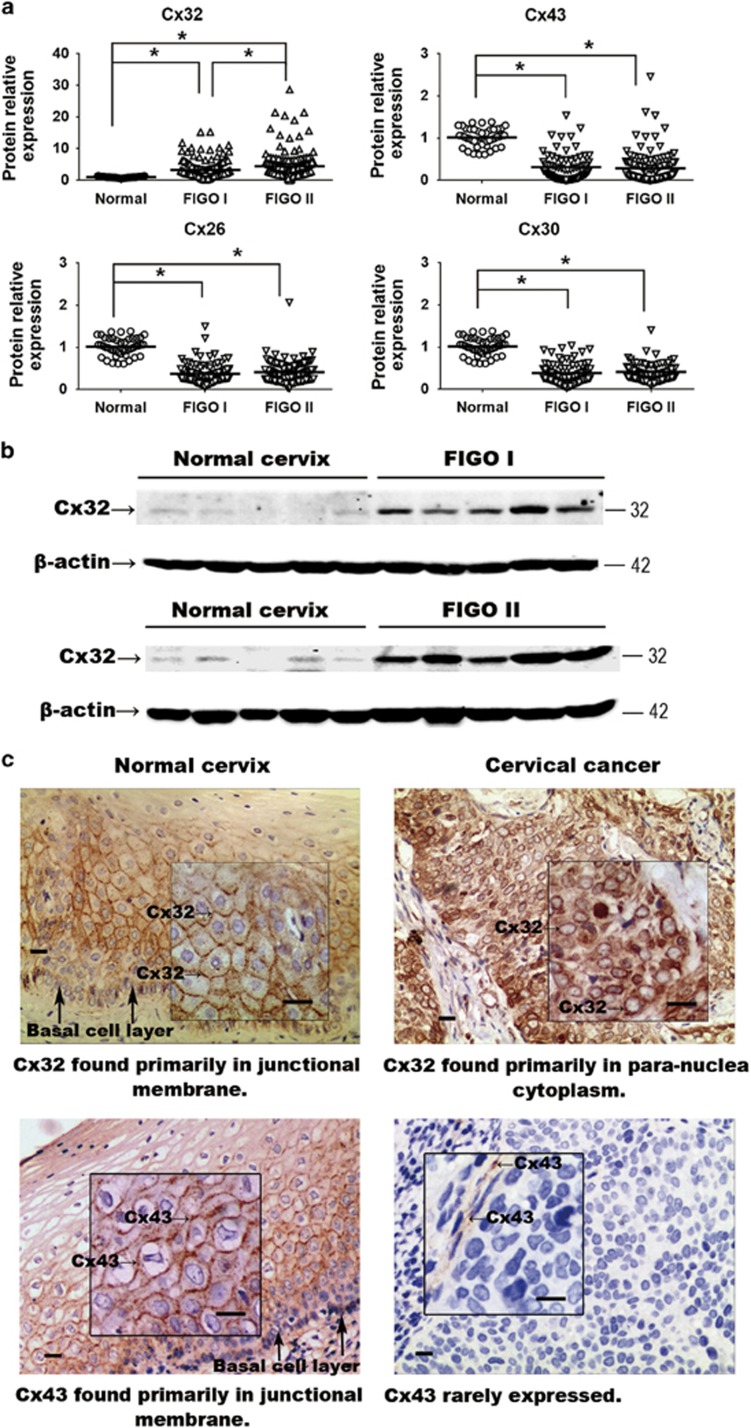
(**a**) Expression of Cx32, Cx26, Cx30 and Cx43 in normal cervix and CaCx samples. Expression of Cx26, Cx30 and Cx43 was decreased in the cancer samples relative to control, while expression of Cx32 was markedly increased. (**b**) Western blots showing that expression of expression of Cx32 correlated with increased FIGO score. Data are shown for five samples in each category (**c**) Immunohistochemistry showing that Cx32 and Cx43 in normal cervix tissue were localized to junctional regions. In CaCx cells, Cx32 aberrantly aggregated in para-nuclear cytoplasm and Cx43 was rarely expressed. (Scale bar: 20 *μ*m). *P<0.05

Expression of Cx26, Cx30, Cx32 and Cx43 was analyzed in human specimens consisting of: Normal cervix (*n*=78), CaCx FIGO stage I (*n*=148), CaCx FIGO stage II (*n*=165). In the CaCx samples, expression of Cx26, Cx30 and Cx43 was significantly reduced (Figure 1a and Table 1). However, the mean of Cx32 expression in CaCx specimens was higher than that in normal cervix specimens, and the degree of upregulation correlated with advanced FIGO stages (Figures 1a, b and Table 1) (FIGO I *versus* Normal, *P*<0.001; FIGO II *versus* Normal, *P*<0.001; FIGO I *versus* FIGO II, *P*=0.0088). To our knowledge, this is the first evidence that Cx32, often considered a tumor suppressive factor, is highly expressed in CaCx tissue, and is specifically upregulated relative to expression in normal cervical tissue.

The cellular localization Cx32 was examined by immunohistochemistry (IHC) in normal cervix (*n*=9) and CaCx (*n*=41) samples. In normal cervix specimens, Cx32 was found essentially exclusively in plasma membrane in 88.8% (8/9) of the samples (Figure 1c, upper left). In dramatic contrast, in Cx32 was found only in the cytoplasm of 92.7% (38/41) of CaCx specimens (Figure 1c, upper right) For comparison, in normal cervix samples, Cx43 was found in plasma membrane in 9/9 cases, but it was detected at very low levels or not at all in CaCx specimens (low levels in 21/41 cases; not detected in 20/41 cases) (Figure 1c, lower), consistent with the western blot results. This downregulation of Cx43 in CaCx specimens was consistent with previous reports.^7, 26^ The above results indicate that Cx32 is specifically upregulated in human CaCx cells and is retained in cytoplasm and so cannot contribute to gap junction formation.

### High expression of Cx32 is correlated with advanced FIGO stage, augmented tumor size and poorer differentiation in human CaCx

On the basis of relative expression levels of Cx32 in the 313 CaCx samples relative to that in controls (mean for all CaCx samples was 3.86 times that in controls), the CaCx data were divided into groups of high expression (>3.86, *n*=196) and low expression (<3.86, *n*=117). A large number of clinical-pathologic variables of the two groups were compared (Table 2). These were: age, ethnic group, FIGO stage, maximum diameter of tumor, lymph nodes metastasis, tumor emboli, whole-layer infiltration, pelvic nerve invasion, and differentiation, recurrence in 3 year and HPV infection. Statistically significant positive correlations were found between high Cx32 expression and FIGO stage (*P*=0.010), tumor maximum diameter (*P*=0.023) and differentiation (*P*=0.22). These results show that high Cx32 expression in CaCx specimens is specifically associated with deteriorated FIGO stage, augmented tumor size and poorer differentiation.

### Overexpression of Cx32 suppressed apoptosis of HeLa and SiHa cells only when GJ function was inhibited

**Figure 2:**
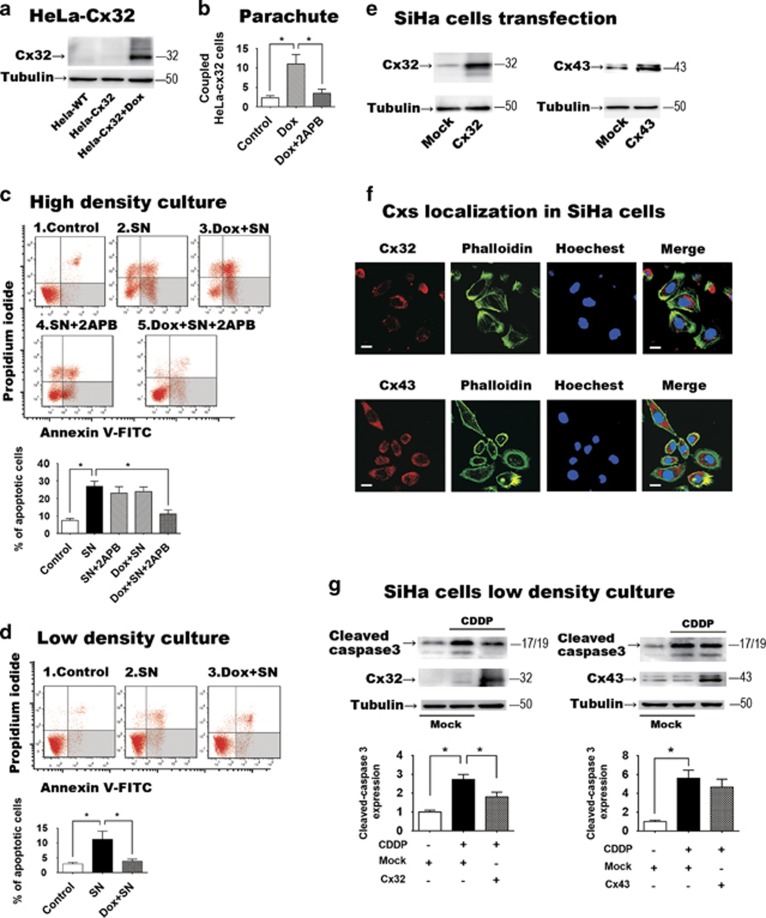
(**a**) Western blot showing induction of Cx32 expression in the HeLa-Cx32 cell line after 48 h doxycycline treatment (*n*=5). (**b**) Demonstration of induction of GJIC following induction of Cx32 expression, and inhibition of GJIC with application of the GJ inhibitor 2APB (50 *μ*M) (*n*=5). GJIC was assessed by parachute assay of dye coupling (**c**) Effects of functional gap junctions on apoptosis induced by streptonigrin (SN, 1 *μ*M). In high-density cultures, which allow formation of GJ, SN-induced apoptosis was suppressed when GJ function was inhibited by 2APB (*n*=7). 2APB had no effect in the absence of induced Cx32 expression. Neither doxycycline (Dox, to induce Cx32 expression) nor 2APB (GJ inhibition) suppressed SN-induced apoptosis when applied alone. (**d**) When GJ formation was physically inhibited by low-density culture, expression of Cx32 suppressed SN-induced apoptosis (*n*=5). (**e**) Transient transfection of SiHa cells induced expression of Cx32 or Cx43 (*n*=3). (**f**) In transfected SiHa cells, both Cx32 and Cx43 mainly localized in cytoplasm (*n*=3, scale bar=10 *μ*m). (**g**) Expression of Cx32, but not Cx43, in SiHa cells suppressed apoptosis induced by cisplatin (CDDP, 10 *μ*M) under low-density culture (*n*=3). Error bar: standard error. *P<0.05; *n*=1 represents an independent cell culture

The above results suggest a correlation between Cx32 expression and advanced tumor stage. To address whether the enhanced Cx32 level was causative, Cx32 expression was induced in the HeLa-Cx32 cell line (see Materials and Methods) with doxycycline. Induction of Cx32 expression was accompanied by increased GJ intercellular communication (GJIC), which was inhibited by the GJ inhibitor 2APB (50 *μ*M) (Figures 2a and b). Treatment of the Cx32-expressing HeLa cells with streptonigrin (SN) (1 *μ*M; 6 h) induced massive apoptosis, consistent with prior work.^29^ However, SN-induced apoptosis was significantly suppressed by treatment of the Cx32-expressing HeLa cells with the GJ inhibitor 2APB (*n*=7, *P*=0.0011). Neither doxycycline nor 2APB alone suppressed apoptosis (Figure 2c). Also, 2APB did not alter SN-induced apoptosis in HeLa wild-type cells (cells not transfected with the tet-ON Cx32 promotor; Supplementary Data).

These results indicate that expression of Cx32 when GJ function is inhibited results in protection against SN-induced apoptosis. To test the above hypothesis, Cx32-expressing HeLa cells were grown in low-density culture, in which there was no opportunity for GJ formation. In this condition as well, in which GJ formation was inhibited physically rather than pharmacologically, increased Cx32 expression also significantly suppressed SN-induced apoptosis (SN *versus* Dox+SN, *P*=0.0379, *n*=5) (Figure 2d). These results demonstrate that Cx32 expression in the absence of functional GJ—which is what is seen in the CaCx clinical samples, based on absence of Cx32 in plasma membrane, suppresses SN-induced apoptosis.

To address whether this anti-apoptotic effect was specific for Cx32, we transiently transfected SiHa cells to express either Cx32 (*n*=3, *P*=0.0042) or Cx43 (*n*=3, *P*=0.031) (Figure 2e). Exogenous gene expressed Cx32 and Cx43 both mainly localized in cytoplasm under confocal microscope in SiHa cells from three independent cultures (Figure 2f). Low-density culture was used to fully inhibit GJ formation between these cells. Following 24 h treatment with cisplatin (10 *μ*M), apoptosis was assessed by expression of cleaved-caspase3. In this assay, expression of Cx32 significantly suppressed cisplatin-induced apoptosis (*P*=0.031, *n*=3) as expected, but expression of Cx43 did not (Figure 2g). These results show both that the anti-apoptotic effect of Cx32 expression in the absence of GJ was not specific to HeLa cells, and that is was specific for Cx32 expression, as it could not be produced by expression of another common connexin, Cx43.

### siRNA knockdown of endogenous Cx32 expression in C-33A cells reversed the anti-apoptotic effect of GJ inhibitors

**Figure 3:**
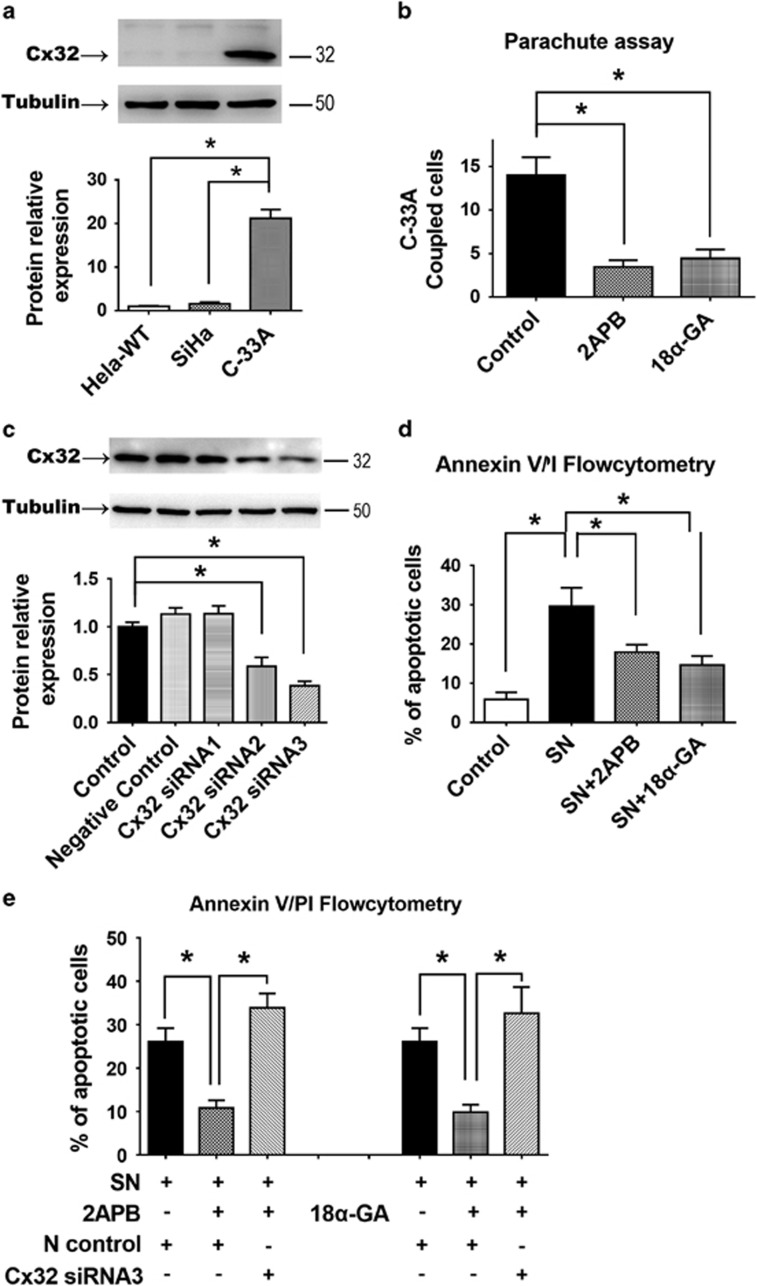
(**a**) C-33A cells endogenously express Cx32, unlike HeLa or SiHa cells (*n*=3). (**b**) The endogenous GJIC of C-33A cells was abolished by gap junction inhibitors 2APB and 18*α*-GA (*n*=3). (**c**) Western blot comparing effectiveness of three siRNAs in reducing expression of Cx32 in C-33A cells. (*n*=3). (**d**) Suppression of SN-induced apoptosis by treatment with 2APB or 18*α*-GA in C-33A cells (*n*=7). (**e**) The anti-apoptotic effect of 2APB and 18*α*-GA in C-33A cells was reversed by Cx32 RNA interference (*n*=5). Error bar: standard error. *P<0.05; *n*=1 represents an independent cell culture

C-33A cells endogenously express Cx32 and form functional GJ, unlike non-transfected HeLa and SiHa cells (*n*=3, *P*<0.001) (Figure 3a). GJIC between C-33A cells is abolished by either 2APB or 18*α*-GA (*n*=3, *P*<0.001) (Figure 3b). Consistent with the previous studies using HeLa and SiHa cells transfected to express Cx32, apoptosis of C-33A cells was induced by 6 h SN (1 *μ*M) treatment, and was suppressed by 2APB or 18*α*-GA (*n*=5, *P*=0.012) (Figure 3d) (2APB and 18-GA did not alter expression of Cx32 in C-33A cells; Supplementary Data). To knockdown the endogenous Cx32 expression in these cells, three different Cx32 siRNA sequences were tested for ability to reduce Cx32 expression; Cx32 siRNA sequence 3 (Cx32-siRNA3) had the greatest efficacy (*n*=3, *P*<0.001) (Figure 3c). Transfection of C-33A cells with Cx32-siRNA3 reversed the anti-apoptotic effects of 2APB (*n*=6, *P*<0.001) and 18*α*-GA (*n*=6, *P*=0.0046) (Figure 3e). This demonstrates that the endogenous Cx32, in the absence of GJ function, was responsible for the anti-apoptotic effect revealed by inhibition of GJ function by 2APB and 18*α*-GA. Thus, in three human cervical cell lines, Cx32 was shown to suppress apoptosis when GJ function or formation is inhibited.

### Cx32 expression up-regulates EGFR and activates its downstream effectors Erk1/2 and Stat3

**Figure 4:**
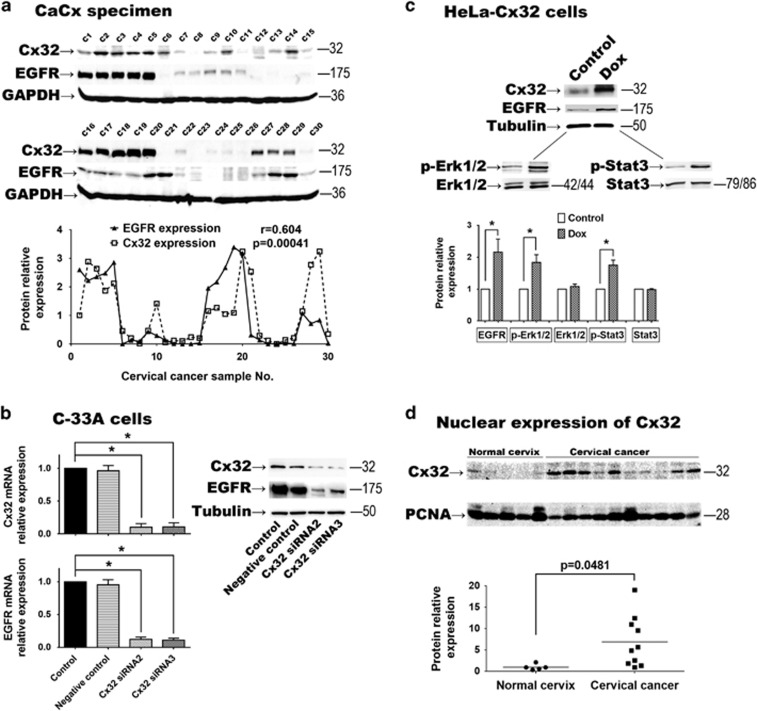
(**a**) In 30 CaCx specimens, expression of EGFR was highly correlated with expression of Cx32 (r=0.604, *P*=0.00041). (**b**) In C-33A cells, when Cx32 expression was inhibited by Cx32 siRNAs, EGFR expression was significantly suppressed (*n*=3). (**c**) In HeLa-Cx32 cells, after induced Cx32 expression, EGFR was significantly increased and its downstream effectors p-Erk1/2 and p-Stat3 were upregulated (*n*=3). (**d**) In nuclear protein samples, Cx32 expression in CaCx cells (*n*=10) was higher than that in normal cervix cells (*n*=5, *P*=0.0152). Error bar: standard error. *P<0.05; *n*=1 represents an independent cell culture; *r*: Pearson correlation coefficient

We found that in 30 cases of CaCx, Cx32 expression was highly correlated with expression of EGFR (*r*=0.604, *P*=0.00041) (Figure 4a). EGFR expression at both protein and mRNA levels were significantly suppressed by Cx32-siRNA3 in C-33A cells (*n*=3, *P*<0.001) (Figure 4b). Analogously, EGFR expression was augmented by induced expression of Cx32 in HeLa-Cx32 cells. Simultaneously, the downstream p-Erk1/2 (*n*=3, *P*=0.040) and p-Stat3 (*n*=3, *P*=0.044) were significantly upregulated (Figure 4c).

To gain insight into how Cx32 expression may affect EGFR expression, nuclear protein samples were obtained from normal and CaCx tissue, and assessed for presence of Cx32. The presence of Cx32 in nuclear protein samples was significantly greater from CaCx (*n*=10) than normal cervix samples (*n*=5) (*P*=0.0152). It has been reported that connexin may directly regulate the transcription of apoptosis regulators via a ‘connexin responsive element’ in the nucleus.^30^ Elevation of nuclear Cx32 could provide a mechanism by which non-GJ Cx32 affects EGFR expression and the anti-apoptotic effect described above.

### Cx32 exerts an anti-apoptotic effect via the EGFR pathway in CaCx cells

**Figure 5:**
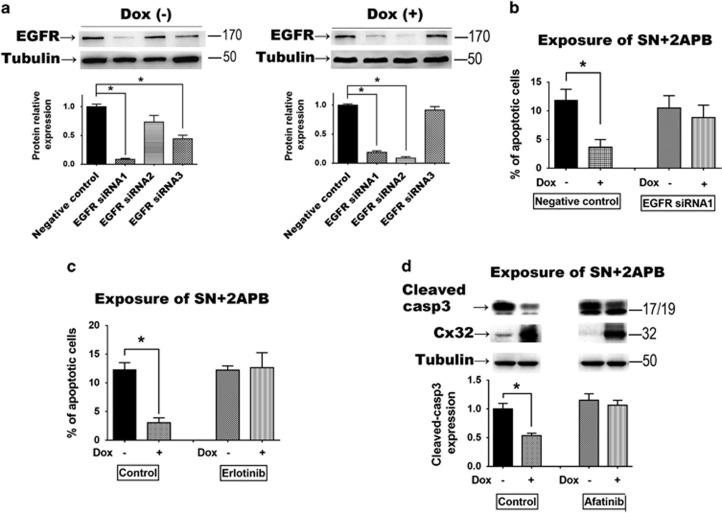
(**a**) In HeLa-Cx32 cells, EGFR siRNA sequence 1 effectively inhibited EGFR expression with and without doxycycline treatment (*n*=3). (**b**) The anti-apoptotic effect of Cx32 expression was reversed by siRNA suppression of EGFR (*n*=3). (**c** and **d**) The anti-apoptotic effect of Cx32 expression was reversed by EGFR signaling inhibitors erlotinib and afatinib (*n*=3). Error bar: standard error. *P<0.05; *n*=1 represents an independent cell culture

Three siRNA sequences targeted to EGFR expression were assessed in HeLa-Cx32 cells. The siRNA sequence 1 (EGFR-siRNA1) effectively suppressed EGFR expression with or without induction of Cx32 expression (*n*=3, *P*<0.001) (Figure 5a). Cx32 expression had the same anti-apoptotic effect under exposure of SN (*n*=3, *P*<0.001) and 2APB (*n*=3, *P*<0.001) as previously (apoptosis was detected by flow cytometry or cleaved-caspase3 expression). However, when EGFR expression was inhibited by EGFR-siRNA1, the anti-apoptotic effect of Cx32 was reversed (Figures 5b–d). The same results were seen with application of two EGFR pathway inhibitors, erlotinib and afatinib. These results show that the EGFR pathway is an essential component of the Cx32-induced anti-apoptotic effect.

## Discussion

Taken together, the results of the present study indicate that the specific upregulation and non-junctional localization of Cx32 in human CaCx cells contributes to tumor growth and chemoresistance. The results also suggest that in the context of chemotherapeutic exposure, Cx32 can have either pro-apoptotic effects (via GJIC) or anti-apoptotic effects (via cytosolic and potentially nuclear localization), with the latter effect involving the EGFR signaling pathway. Thus, the balance between junctional and cytosolic localization of Cx32 is likely to determine the overall effect.

Cx32 was specifically upregulated in human CaCx specimens, unlike three other connexins, which were downregulated. Elevated Cx32 expression was associated with deteriorated FIGO stage, augmented tumor size and poorer differentiation. To our knowledge, this is the first clinical-pathological evidence showing that a Cx is highly expressed in CaCx cells. Previous reports indicated that other Cx are downregulated in CaCx,^6, 28, 31^ a finding reproduced here. However, the highly elevated Cx32 expression and its distribution in cytoplasm of CaCx cells have not been previously reported. Our *in vitro* studies of the effects of strong Cx32 expression in the absence of GJIC indicate that this key finding has important consequences for tumor biology and therapy: In the *in vitro* studies, Cx32 suppressed streptonigrin/cisplatin-induced apoptosis only after GJ function was inhibited, either pharmacologically or physically. We speculate that due to the non-junctional localization of Cx32, the anti-apoptotic effect is dominant in CaCx tissue. This finding can be a comprehensive explanation of the tumor promoting effect of Cx32 in our clinical-pathological studies. The reason for the non-junctional localization of the Cx32 in the CaCx cells remains unknown at this time, but may offer a point of therapeutic intervention.

Building on the novel finding of specific upregulation and non-junctional localization of Cx32 in the clinical samples, *in vitro* studies were utilized to comprehensively explore the effects of these parameters on chemoresistance and growth. Three human CaCx cell lines were used, each expressing (or not expressing) Cx32 by a different mechanism (HeLa-Cx32: stable transfection with an inducible promotor; SiHa: transient transfection to express Cx32 or Cx43; C-33A: endogenous expression of Cx32 which could be suppressed by siRNA). These systems allowed us to manipulate Cx32 expression both up and down, in transfected and endogenously expressing in CaCx cells, and to independently manipulate GJ formation/function by pharmacological and physical means. The results were consistent across all manipulations and systems: the presence of Cx32 not involved in GJs exerted a protective effect against chemotherapeutic agents.

The applied anti-tumor drugs induce apoptosis by an intrinsic pathway.^32, 33^ GJs are well-known to facilitate intrinsic apoptosis via ‘death signal’ transmission among cancer cells.^9, 34, 35^ In the present study, Cx32 expression did not enhance streptonigrin-induced apoptosis in human CaCx cells, even though it suppressed apoptosis after 2APB inhibited the GJ. We infer that prior to GJ inhibition by 2APB, Cx32 expression exerted two opposing effects, a toxic bystander effect mediated by GJ, and an anti-apoptotic effect medicated by the intracellular accumulation of Cx32. In this case, it appears that these two effects counteracted each other to balance the change of apoptosis in ‘Dox+SN’ group, and that once the GJ-mediated toxic effect was eliminated by 2APB, the protective effect was revealed. Notably, the anti-apoptotic effect of Cx32 in CaCx cells was not reproduced by forced expression of Cx43, showing that the effect is specific for Cx32, the only connexin found to be upregulated in the CaCx tissue.

**Figure 6:**
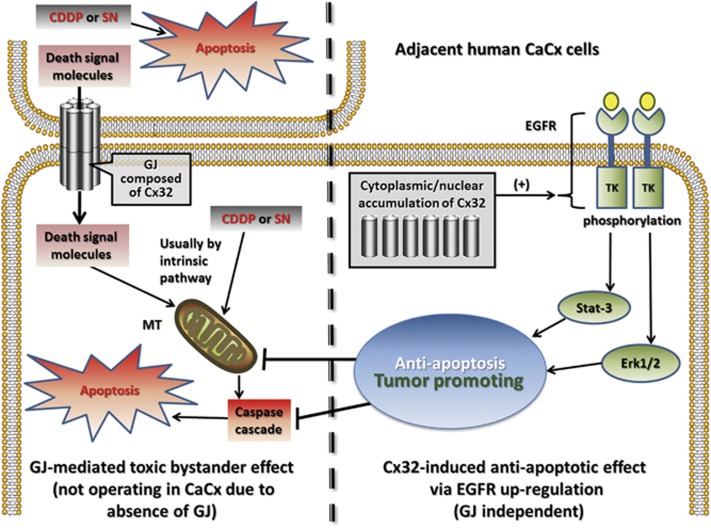
Diagram of the inferred Cx32 anti-apoptotic mechanism. *in vitro*, Cx32 suppressed SN/CDDP-induced apoptosis only after GJ function was inhibited, either pharmacologically or physically. This suggests that upregulated expression of Cx32 *per se* has an anti-apoptotic effect, in contrast to its GJ-dependent pro-apoptotic effect, with exposure to these chemotherapeutic agents. Because of the upregulated Cx32 and its non-junctional localization in CaCx, the anti-apoptotic effect of Cx32 may dominate in cancer cells *in vivo*. Further, clinical-pathological data and *in vitro* findings indicate that the anti-apoptotic and tumor promoting effects involve the EGFR pathway in human CaCx cells. CDDP, Cisplatin; CaCx, cervical cancer; MT, mitochondria; SN, streptonigrin; TK, tyrosine kinase

In other reports, independent of GJ, the ability of Cx to affect apoptosis varies for different Cx and different tissues.^36^ Although Cx composes GJ, recent studies have shown a variety of non-GJ-mediated effects of Cx in cancer cells.^37, 38^ We found that Cx32 was more likely to be found in cell nuclei in CaCx cells than in controls. This result is in line with Dang’s report regarding Cx43 nuclear localization in nucleus and its regulation of cancer cell viability.^39^ It was reported that in the nucleus Cx can interact with ‘connexin responsive element’ (CxRE) to modulate transcription of apoptosis-related proteins.^30, 40^ Sulkowski and colleagues found that expression of Cx26 correlated with expression of insulin-like-growth factor I receptor (IGF-IR) in human colorectal cancer.^41^ Munoz also reported an interaction between Cx43 and EGFR signaling to induce chemoresistance against temozolomide in glioblastoma-multiforme cells.^21^ In line with the above evidence, our study demonstrates a relationship between Cx32 and EGFR in human specimens. In our pathological and *in vitro* studies, Cx32 was shown to be an active regulator of the EGFR pathway, which was necessary for its anti-apoptotic effects. The experiments using EGFR siRNA and EGFR pathway inhibitors show that the EGFR pathway is a critical element in Cx32 suppression of apoptosis. On this basis we propose that the EGFR pathway is a key mediator of the chemoprotective effects of non-junctional Cx32 in CaCx cells. Thus, all evidence-based findings in current study were summarized into an inferred diagram (Figure 6), and a serial of Cx32-related mechanisms were indicated.

Although Cx32 tumor promoting effect was well supported by both clinical pathology and *in vitro* data, its role in CaCx cell’s proliferation and metastasis is unclear. There is still a logical gap between *in vitro* apoptosis suppression and deteriorated prognostic variables to fill. A serial of xenograft animal model based experiments are preferable to be utilized for further study.

In conclusion, Cx32, traditionally tumor suppressive protein, was shown to be tumor protective against chemotherapy *in vitro* if it is prevented from forming GJs. This finding indicates that Cx32 can be a promising tumor marker to predict chemotherapeutic sensitivity of CaCx. Cross-regional prospective clinical trials are required to test this idea. Moreover, as Cx32 expression is elevated in many cases of CaCx, it might be feasible to therapeutically recover their GJIC to suppress tumor growth by manipulating the trafficking pathway. Further research focusing on recovering the Cx trafficking system in CaCx cell is needed.

## Materials and Methods

### Human cervix specimens and clinical data

Staging of CaCx was following the reported Federation International of Gynecology and Obstetrics (FIGO) system.^42^ Human cervix tissue samples were taken from patients who underwent total hysterectomy from 2012 to 2014 for treatment of CaCx (FIGO stage I, *n*=148; FIGO stage II, *n*=165) and benign multiple uterine fibroids (*n*=78; used as normal cervix controls). After hysterectomy, cervix specimens were kept in liquid nitrogen for protein extraction or paraffin embedding. Clinical variables including age, ethnic group, maximum diameter of tumor, FIGO staging, lymph node metastasis, tumor emboli, differentiation, whole-layer infiltration, pelvic nerve invasion, recurrence and HPV infection were recorded for at least 2 years of following up. Expression and intracellular distribution of Cx26, Cx30, Cx32 and Cx43 were detected by western blot and immunohistochemistry. The study was approved by the Research Committee of Ethics in the Affiliated Cancer Hospital of Xinjiang Medical University.

### Cell culture and authentication

As previously described,^43^ HeLa-Cx32 is a stable transgenic cell line with a tetracycline-inducible promoter, capable of constitutively expressing Cx32 when treated with doxycycline (Calbiochem, San Diego, CA, USA). C-33A and SiHa cells were purchased from American Type Culture Collection (Manassas, VA, USA). Cells were cultured in DMEM (C-33A in MEM) with 10% fetal bovine serum at 37 °C, 5% CO_2_, in a humidified incubator. As previously described, Hela-Cx32 cells were grown in above medium with 100 *μ*g/ml of G418 (Calbiochem) and 200 *μ*g/ml of hygromycin B (Calbiochem). HeLa-Cx32 robustly expressed Cx32 after 48 h of doxycycline (1 *μ*g) treatment. For low-density cultures, 1 × 10^5^ cells were seeded in a 150 mm dish to physically inhibit gap junction formation. For high-density cultures, 1 × 10^5^ cells seeded in a well of 35 mm well plate to allow gap junction formation. The tool drugs including Cisplatin (CDDP), streptonigrin (SN), 18-*α*-glycyrrhetinic acid (18*α*-GA), 2-aminoethoxydiphenyl-borate (2APB) were purchased from Sigma-Aldrich (St. Louis, MO, USA), whereas erlotinib and afatinib were from Selleck Chemicals (Houston, TX, USA).

Three CaCx cell lines (HeLa, SiHa, C-33A) were authenticated by short tandem repeat (STR) polymorphism analysis. DNA samples of cells were extracted and amplified with STR Multi-amplification Kit (PowerPlexTM 16 HS System, Promega Corporation, Madison, WI, USA) and assayed with ABI 3100 DNA Analyzer (Applied Biosystems, Thermofisher Scientific, Waltham, MA, USA). STR profiles of samples matched that of respective cell lines from ATCC and DSMZ data bank. No contamination of other human cell lines or other species was found in result.

### DNA plasmid transfection and siRNA interference

Plasmid vectors of Cx32 (Product No. EX-A0514-M02-5) and Cx43 (Product No. EX-A0334-M02-5) were constructed by Genecopoeia (Rockville, MD, USA). Before DNA plasmid transfection, SiHa cells were seeded in 6 well plates and grown to 80% confluence. Following the manufacturer’s instructions, each 10 *μ*l Lipofectamine 2000 (Invitrogen, Carlsbad, CA, USA) was mixed with DNA plasmid (4 *μ*g). The Lipofectamine-DNA compound was added to cell medium and kept for 6 h before change to normal medium. 48 h later, expression of Cx32 or Cx43 was assessed by real-time-qPCR and western blotting.

For siRNA transfection, C-33A or Hela-Cx32 cells were grown to 30–50% confluence. Then the cells were transfected with respective siRNAs (Ribbon, Guangzhou, China) at a concentration of 50 nM using Entranster R4000 transfection reagent (Engreen Biosystem, Beijing, China) according to the manufacturer’s protocol. After 48 h, suppression of Cx32 expression was detected by real-time-qPCR and western blotting. The sequences for the synthetic siRNAs Targeting Cx32 (siCx32) were as follows:

siCx32_1: 5′-CCGGCATTCTACTGCCATT-3′,

siCx32_2: 5′-GGCTCACCAGCAACACATA-3′,

siCx32_3: 5′-GCAACAGCGTTTGCTATGA-3′.

The sequences for the synthetic siRNAs targeting EGFR (siEGFR) were as follows:

siEGFR_1: 5′-GGCTGGTTATGTCCTCATT-3′,

siEGFR_2: 5′-CCTTAGCAGTCTTATCTAA-3′,

siEGFR_3: 5′-GGAACTGGATATTCTGAAA-3′.

### Extraction of total and nuclear protein

For total protein extraction, homogenized tissue (50–100 mg) were rinsed with PBS and treated in ice-cold lysis-buffer. After ultra-sonication, the lysate solutions were centrifuged at 12 000 rcf for 30 min at 4 °C. For nuclear protein extraction, a commercial kit (NE-PER Nuclear and Cytoplasmic Extraction Reagents, ThermoScientific, MA, USA) was applied following the manufacturer’s instructions and previous method.^44^ Thus, the extracted supernatant (nucleoprotein) was moved into a pre-cooled tube after centrifugation (12 000 rcf) at 4 °C for 20 min. Bio-Rad protein assay kit (Hercules, CA, USA) was used to measure protein concentration.

### Western blot analysis

An equal amount (20 *μ*g) of each protein sample was added into SDS-PAGE gel for electrophoresis, and then transferred to a nitrocellulose membrane. The membranes were blocked with 5% (w/v) skimmed milk in wash buffer (TBS and 0.05% Tween 20) for 1 h. The respective primary antibodies were incubated with membranes overnight at 4 °C. GAPDH, *β*-tubulin and *β*-actin were loading control markers for total protein and PCNA was that for nuclear protein. Primary antibodies against Cx32, Cx43, Cx26, Cx30 were purchased from Sigma-Aldrich (Respective product ID: C6344, C8093, SAB2500466, SAB2104321). Other primary antibodies against EGFR, ERK1/2, p-ERK1/2(Thr202/Tyr204), STAT3, p-STAT3 (Tyr705) and cleaved-caspase3 were obtained from Cell Signaling Technology (Danvers, MA, USA). Antibody dilution of Cx26, Cx30, Cx32, EGFR, ERK1/2, p-ERK1/2, STAT3, Caspase3, p-STAT3 was 1:1000, dilution of Cx43 antibody was 1:5000, dilution of GAPDH, *β*-tubulin and *β*-actin was 1:10 000. The secondary antibody was incubated with membrane for 1 h at room temperature at a dilution half that of the primary antibodies. The immune-reactive bands were visualized by Amersham ECL Plus Western Blotting Detection Kit (GE Healthcare, Piscataway, NJ, USA), scanned and quantified by ImageQuant LAS 4000 and its associated software (GE). When processing band density data from human specimens and *in vitro* samples, ratio of target biomarker to respective loading control was calculated. Mean of the ratio from control bands were defined as ‘1’ and fold changes of every sample’s ratio to the mean were the finalized data.

### Immunohistochemistry and immunofluorescence analysis

Normal cervix or CaCx tissues from patients were fixed with buffered formalin at 4 °C overnight, processed through graded ethanol solutions, and embedded in paraffin blocks. Tissue samples were sectioned to 10 *μ*m thick slices. The sections were dewaxed and dehydrated in gradient ethanol to water then autoclaved at 121 °C for 10 min in 100 mM citrate buffer (pH 6) for retrieving antigens prior to staining. The sections were treated with 3% H_2_O_2_ for 30 min and 10% goat serum for 1 h at room temperature to block endogenous nonspecific reactivity. Primary antibodies of Cx32 and Cx43 were incubated with sections at dilution of 1:200 and 4 °C overnight before biotinylated secondary-antibodies were applied to label antigens for 30 min at 37 °C. The specific antigens were visualized by using the Vectastain ABC kit (Vector Laboratories, Burlingame, CA, USA) according to the manufacturer’s protocol. Hematoxylin was used to counter-stain on the sections. Distribution of Cx32 and Cx43 was observed and captured under Olympus BX-51 microscope (Olympus, Tokyo, Japan).

For immunofluorescence imaging, the transient transfected SiHa cells were cultured in 16-well plate for 24 h. After 3 times PBS rinse, the cells were fixed with 4% paraformaldehyde for 30 min. Incubate with 0.1% Triton X100 for 20 min, then block by 2% BSA for 30 min under room temperature. Thus, primary antibody (Cx32 and Cx43, dilute to 1:200) was applied and incubated overnight in 4 °C. After PBS rinse, incubated with Alexa Fluor555 conjugated secondary antibody (from ThermoFisher Scientific) (1:400) for 1 h under room temperature in dark hood. Phalloidin (5 *μ*g/ml) and hoechst (1 *μ*g/ml) were applied sequentially for actin and nuclear staining. After fully PBS rinse, immunofluorescence images of cells were captured under confocal microscope (LSM710, Carl Zeiss Jena, Germany).

### Flow cytometry apoptosis detection assay

Hela-Cx32 cells were seeded in six-well plates and cultured with or without doxycycline for 48 h (80–100% confluence). The SiHa cells were transfected with plasmid of Cx32, Cx43 or vector for 48 h; C-33A cells were transfected with siRNA Cx32 for 48 h. Cells were incubated with SN (1 *μ*M) for 6 h or Cisplatin (10 *μ*M) for 24 h. Afterward, the cells were washed three times in cold PBS and then trypsinized and harvested. Cells were re-suspended in binding buffer and after double staining with Annexin V-FITC and propidium iodide (PI) using the Annexin V-FITC apoptosis detection kit (Biotool, Houston, TX, USA) according to the manufacturer's protocol. The data were immediately analyzed by flow cytometry using the Expo32 Software (Beckman Coulter, 250 S. Kraemer Boulevard Brea, CA, USA) for determination of apoptotic cells.

### Parachute dye-coupling assay

This assay was used to assess GJIC function as previously described.^45^ Cells were cultured to 80–90% confluence in 12-well dishes. Calcein-AM and CM-DiI were bought from Invitrogen. Donor cells were double-labeled with Calcein-AM (green fluorescence, GJ permeable) and CM-DiI (red fluorescence, non-permeable) for 30 min at 37 °C. Cells were rinsed, trypsinized and seeded onto the receiver cells at a 1:150 donor/receiver ratio then incubated for 4 h at 37 °C. Using a fluorescence microscope (Olympus IX71, Tokyo, Japan), the average number of receiver cells (green fluorescence) around every donor cell (both green and red fluorescence) was recorded as an index of GJIC function.

### Real-time-qPCR

The total RNA was extracted using the Hipure Total RNA Kits (Magen, Guangzhou, Guangdong, China) according to the manufacturer's instructions. The collected RNA was reverse-transcribed using the reverse transcription kit (Transgen Biotech, Beijing, China) and the resulting cDNA was subjected to qPCR and RT-PCR reactions performed in a final volume of 20 *μ*l using the quantitative real-time PCR kit (Transgen Biotech) according to the manufacturers’ instructions. All primer sequences in this study were acquired from PrimerBank (Massachusetts General Hospital, Boston, MA, USA),^46^ including: Cx32 (PrimerBank ID 195222738c1), Cx43 (PrimerBank ID 122939163c1) and EGFR (PrimerBank ID 41327735c1). Resulting CT value for every target gene in every sample was normalized to the respective value of GAPDH to acquire the relative expression data.

### Statistical analysis

Every *in vitro* experiment was performed with a minimum of three independent cell cultures. Statistical analysis utilized Graphpad Prism 6.0 software. Parametric clinical variables are presented as mean and 95% confidence interval. Parametric data were analyzed by one-way ANOVA or student's *t*-test, nonparametric data were analyzed by Fisher’s exact test, and correlation between Cx32 and EGFR expression was analyzed by Pearson’s correlation analysis. Statistical significance was defined as *P*<0.05 and all tests were two sided.

### Data availability

All supporting data in this work are also available in figshare. URL: https://figshare.com/s/575ba44ead4c94daccbb DOI: 10.6084/m9.figshare.4543066

## Figures and Tables

**Table 1:** Protein expression of four connexin isotypes in normal cervix and different FIGO stage cervical cancer specimens

	**Normal cervix** **(*n*=78)**	**FIGO I** **(*n*=148)**	**FIGO II** **(*n*=165)**
Cx32	1.015±0.213	3.262±2.915^a^	4.434±4.450^a^^b^
Cx43	1.017±0.212	0.316±0.319^a^	0.285±0.357^a^
Cx26	1.016±0.212	0.376±0.199^a^	0.413±0.222^a^
Cx30	1.016±0.213	0.389±0.221^a^	0.414±0.211^a^

Abbreviation: FIGO, Federation International of Gynecology and Obstetrics

a Comparing with Normal cervix, *P*<0.05

b Comparing with FIGO I, *P*<0.05

P.S.: Data were presented as mean±S.D.

**Table 2:** Relationship between Cx32 expression and clinical variables

	**Low expression** **(<3.86, *n*=196)**	**High expression** **(>3.86, *n*=117)**	***P-*** **value**
Age	47.79 (46.49, 49.09)	50.16 (48.59, 51.73)	N.S.
*Ethnic*			
Han	108 (34.5%)	58 (18.5%)	N.S.
Uyghur	88 (28.1%)	59 (18.8%)	
			
*FIGO stage*			
I	104 (33.2%)	44 (14.1%)	**0.010**
II	92 (29.4%)	73 (23.3%)	
Maximum diameter of tumor (cm)	3.08 (2.88, 3.29)	4.04 (3.62, 4.46)	**0.023**
			
*Lymph node metastasis*			
Positive	41 (13.1%)	22 (7%)	N.S.
Negative	155 (49.5%)	95 (30.4%)	
			
*Tumor emboli*			
Positive	48 (15.3%)	37 (11.8%)	N.S.
Negative	148 (47.3%)	80 (25.6%)	
			
*Differentiation*			
Poorly	50 (16%)	45 (14.4%)	**0.022**
Moderately/well	146 (46.6%)	72 (23%)	
			
*Whole-layer infiltration*			
Positive	55 (17.6%)	43 (13.7%)	N.S.
Negative	141 (45%)	74 (23.6%)	
			
*Pelvic nerve invasion*			
Positive	8 (2.6%)	5 (1.6%)	N.S.
Negative	188 (60.1%)	112 (35.8%)	
			
*Recurrence in 2 years*			
Positive	1 (0.3%)	2 (0.6%)	N.S.
Negative	195 (62.3%)	115 (36.7%)	
			
*HPV infection*			
Positive	144 (46%)	86 (27.5%)	N.S.
Negative	17 (5.4%)	13 (4.2%)	
Not detected	35 (11.2%)	18 (5.8%)	

Abbreviation: N.S., not significant

P.S.: Mean of Cx32 relative expression: 3.86, total 313 cases. Parametric data were presented as mean and 95% confidence interval
