# Supplementary figures and images for: Non-junctional Cx32 mediates anti-apoptotic and pro-tumor effects via epidermal growth factor receptor in human cervical cancer cells
Source: Cell Death Dis. 2017 May 11;8(5):e2773–. doi: 10.1038/cddis.2017.183 (PMC5520707; doi:10.1038/cddis.2017.183)

# Supplemental figure

## HeLa-wt cells

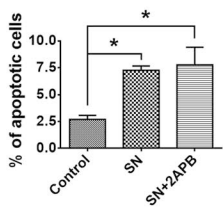

Supplement: Supplementary Figure 1 [file cddis2017183x1.pdf]
