# Supplementary material for: Non-junctional Cx32 mediates anti-apoptotic and pro-tumor effects via epidermal growth factor receptor in human cervical cancer cells
Source: Cell Death Dis. 2017 May 11;8(5):e2773–. doi: 10.1038/cddis.2017.183 (PMC5520707; doi:10.1038/cddis.2017.183)
Supplement: Supplementary Figure 1 Legend [file cddis2017183x2.docx]

**Supplemental Figure Legend**

**Supplemental Figure.** In HeLa wild type cells (parental; not transfected with the inducible Cx32 expression vector), SN (1μM, 6hr) induced significant apoptosis (n=4), whereas 2APB did not alter apoptosis. *: p<0.01
